# Immunomodulation in Primary Immune Thrombocytopenia: A Possible Role of the Fc Fragment of Romiplostim?

**DOI:** 10.3389/fimmu.2019.01196

**Published:** 2019-06-04

**Authors:** Alexandra Schifferli, Falk Nimmerjahn, Thomas Kühne

**Affiliations:** ^1^Department of Hematology/Oncology, University Children's Hospital Basel, Basel, Switzerland; ^2^Department of Biology, Institute of Genetics, University of Erlangen-Nürnberg, Erlangen, Germany

**Keywords:** ITP, immunomodulation, thrombopoietin receptor agonist, Fc-Fragment, peptibody, romiplostim, Fc fusion protein

## Abstract

Fc fusion proteins and Fc fusion peptides or peptibodies are chimeric molecules composed of an active pharmacological protein or peptide and the Fc fragment of an immunoglobulin. The primary aim of this drug construct is to prolong the half-life of the active component. This molecular architecture is seen in drugs, such as etanercept, romiplostim, and the recombinant factor VIII (efmoroctocog alfa). A considerable number of Fc fusion proteins and peptibodies are currently in pre-clinical and clinical development. The isolated effect of the Fc fragment has been studied intensively during last years, but is still poorly understood in the clinical setting and in relation with the active drug and underlying disease. In this short review, we will propose new hypotheses of possible immunomodulatory functions of the Fc fragment of romiplostim in patients with primary immune thrombocytopenia.

## Introduction

Fc fusion proteins and peptides are drugs designed with the aim to prolong the half-life of the effector molecule (1, 2). The Fc domain itself may harbor potential features that may be explored, particularly its immunomodulatory properties that could interfere with the immune system and restitute immune tolerance. In the last years, *in vitro* models and mouse studies brought some answers on selected interactions. Based on observations with the recombinant factor VIII (rFVIIIFc, efmoroctocog alfa) there is potential to assess the properties and effects of the Fc domain in the whole immunological complexity of the patient and in the clinical setting. In this short review, we will summarize the potentially immunomodulatory functions of the Fc fragment, which could explain some effects of the peptibody romiplostim.

## Clinical Background

Primary immune thrombocytopenia (ITP) is an autoantibody-mediated platelet disorder with both accelerated platelet destruction and impaired megakaryopoiesis. The immune dysregulation in ITP involves many levels of the innate and adaptive immune system. The mechanisms underlying the development of autoimmunity are multiple, most of which involve defects in regulatory mechanisms, cytokine production, and signaling pathways. The pathological production of autoantibodies seems to be supported by an imbalance in the T-cell subsets. Patients with ITP harbor a shift toward Th1 and Th17 cells reflecting a feature of many autoimmune diseases (3–7). Moreover, it is known that there is a decrease in regulatory T and B cells resulting in loss of tolerance (8, 9).

**Figure 1:**
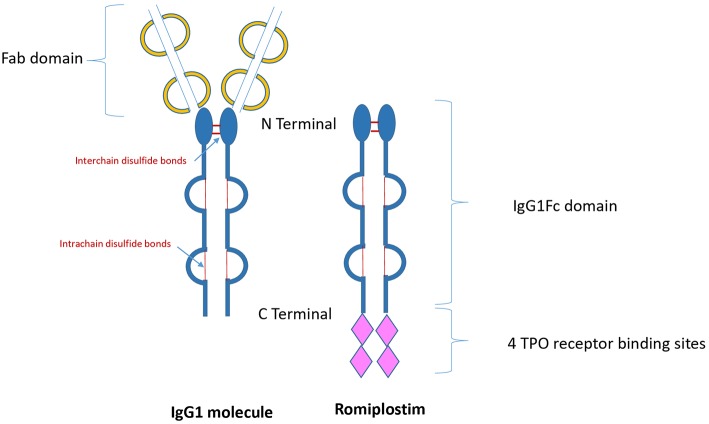
Structure of immunoglobulin and romiplostim. TPO, Thrombopoietin; IgG1, Immunoglobulin G1.

Thrombopoietin receptor agonists (TPO-RAs) are drugs that have been designed to stimulate platelet production via stimulation of the c-mpl receptor. Since 2008, the US Food and Drug Administration approved both TPO-RAs romiplostim and eltrombopag for the treatment of patients with chronic ITP (10–12). Romiplostim is composed of a carrier molecule-IgG1 Fc portion- to which are attached four 14-amino acid TPO peptides (peptibody), which stimulate the TPO receptor (c-mpl) by binding to the extracytoplasmic domain similar to that of endogenous thrombopoietin (Figure 1). Elthrombopag is a non-peptide small molecular weight TPO-RA, which is orally available. The binding site at the c-mpl receptor is different to that of romiplostim (13).

Data suggest that both TPO-RAs romiplostim and eltrombopag may induce tolerogenic mechanisms in ITP (14–16). In the last years increasing data on long-term remission after discontinuation of TPO-RAs in chronic adult ITP have been published. Case reports, retrospective analysis, and clinical trials reported sustained platelet counts above 100 × 10^9^/L after stopping treatment in up to 30% of the patients (17–19). So far, no clear predictors of remission could be identified in adults; however, a number of these patients had ITP for <1 year (persistent ITP) and received TPO-RAs for a short time period of <1 year. This hypothesis was verified in a prospective trial in adult patients with ITP lasting for <6 months; sustained remission was observed in nearly one third of patients who were treated with romiplostim with a median time (range) to onset of 27 (range 6–57) weeks (18). Recently a romiplostim extension study in children with chronic ITP demonstrated treatment-free responses (platelet count ≥50 × 10^9^/L) in 23% of patients withholding all platelet-enhancing drugs. Younger age at first dose and a platelet count >200 × 10^9^/L in the first 4 weeks of treatment were associated with sustained response lasting ≥6 months (20).

The pathophysiological mechanism of this unforeseen sustained response is currently discussed in the literature (14–16, 21) and is investigated in clinical trials and laboratory research projects, such as in the iROM study (clinicaltrial.gov, NCT02760251). Various mechanisms may co-exist supporting and amplifying the tolerogenic effects of TPO-RAs (21), such as the platelet mass itself, elevation of TGF-beta, and the ability of platelets to effectively cross-present peptide antigens to CD8+T cells (22). It appears that this process is conferred upon platelets by their parent cells, i.e., TPO-RA-stimulated megakaryocytes (23). Spontaneous improvement or even resolution of the disease could also be a possible explanation in some of the patients.

## Pharmacological Background of Fc Fusion Proteins and Peptides

Fc-fusion proteins and Fc-fusion peptides (peptibodies) are molecules that are composed of an immunoglobulin (Ig) Fc fragment that is grafted to a protein or peptide of interest (e.g., receptors, cytokines, ligands, and enzymes) (1). Etanercept, a recombinant human tumor necrosis factor (TNF) receptor-fused to a Fc domain, was the first Fc-Fusion protein approved in USA in 1998. Ten years later romiplostim was the first peptibody being approved by the FDA. Other examples of peptibodies are trebananib and dulaglutide (1). Peptibodies are mainly produced chemically through recombinant DNA technology. Various peptibodies are currently in preclinical and clinical development. The development of peptibodies is challenging and is not comparable to the design and manufacture of antibody-drug conjugates (ADC), such as gemtuzumab ozogaminin or brentuximab vedotin.

The primary aim of the Fc fusion is to extend the half-life of the active protein or peptide. Different mechanisms and properties of the Fc fragment are involved and are known to improve pharmacokinetics, bioavailability and biodistribution of the substance (1, 2, 24–26). The Fc fragment improves directly the stability and availability of the drug; in addition, it reduces the renal clearance (glomerular filtration) because of the bigger size of the molecule. Above all, the Fc domain confers indirectly a prolonged circulation of the drug through the neonatal Fc receptor (FcRn), resulting in recycling the fusion protein or peptibody (27). The FcRn salvage pathway is probably the main mode of action, which leads to a prolongation of the half–life, a mode of action that is well-known to protect immunoglobulin G and albumin from catabolism.

The Fc fragment of the Fc fusion products corresponds to the Fc region of immunoglobulin G and can be composed of IgG1, IgG2, IgG3, or IgG4, most commonly the IgG1 protein is used (24–26). The structure of the IgG subtype affects many properties of the fusion drug. Most important is the variable interaction with the FcRn. It is known that IgG3 has a lower binding affinity and therefore a shorter half-life than expected. Moreover, the IgG subtypes show different immunological activities, due to different binding affinities to the family of Fcγ receptors (28).

To date, the potential immunological activity of the Fc fragment is evaluated in only few Fc-fusion drugs (29). However, the Fc domain may be of importance and confers additional biological, pharmacological, and therapeutic properties to the final drug, such as enhanced Fc-mediated effector functions or the stimulation of immune mechanisms, and will be discussed here.

## Immunological Aspects of the Fc Fragment

There are a number of potential mechanisms by which the Fc region may have immunomodulatory effects (30–34). The first observations originate from Borel et al. (35) and later from Zambidis et al. (36) and showed that coupling of haptens or epitopes to an IgG domain can induce targeted tolerance. Since then it is supposed that the Fc fragment may confer tolerogenic effects on potentially immunogenic drugs, e.g., probably for etanercept (Enbrel®). The immunomodulatory properties are thought to be mostly mediated by the following 3 mechanisms:

First, the specific binding of the Fc region with the FcRn is of importance. It has become clear more recently that the FcRn is much more than a simple recycling receptor and may play an important role in immune surveillance and regulation of immune response. FcRn plays a fundamental role in antigen presentation pathways by MHC I and MHC II (37, 38).

Second, the interaction with the family of Fcγ receptors plays a complex role. This class of receptors exhibit several activating (FcγRI, FcγRIIa) and one inhibitory receptor (FcγRIIb) (28). Here some few examples of the interaction with the Fcγ receptors will be mentioned: on B cells, the inhibitory FcγRIIb is essential for the maintenance of humoral tolerance; and on dendritic cells FcγRIIb regulates cell maturation, antigen presentation, and T-cell as well as regulatory T cell (Treg) expansion, thereby indirectly controlling the cellular immune response. However, not only romiplostim seems to interact or modify the FcγR balance in patients with ITP. A study with eltrombopag showed a decrease in FcγRI and elevation in FcγRIIb on monocytes and a functional decrease in phagocytic capacity (16). Another mechanism of immune modulation could be that selected sugar residues in the sugar moiety attached to the IgG-Fc portion may directly bind IgG to a set of type II Fc-receptors, expressed on innate immune cells, B cells and dendritic cells. For example, IgG glycovariants with terminal sialic acid residues were shown to dampen immune responses and expand regulatory T cells via binding to DCIR and DC-SIGN, which can induce Treg expansion (39, 40). It has to be noted, however, that romiplostim is currently produced in bacterial cultures and not in eukaryotic cell lines. Thus, the Fc-portion of romiplostim will not be glycosylated and the interaction of aglycosylated Fc-domains with classical FcgRs will be strongly reduced. In contrast, FcRn binding will not be affected by the sugar moiety in the IgG Fc-portion.

Third, De Groot et al identified in 2008 two epitopes of the Fc fragments capable of activating Tregs, and named them “Tregitopes” (33, 41). Tregitopes sequences are processed and presented by the MHC II receptor on antigen-presenting cells and seem to induce Tregs and promote tolerance in mice studies (41, 42). It is well known that patients with ITP have reduced functional Tregs (8) and a T cell imbalance toward the Th1 subset (5, 7). Whether the fusion of peptides to the CH3 IgG domain impacts the correct processing and presentation of the Tregitopes present in the romiplostim is unknown and would need further investigations. However, Tregitopes could be a possible mechanism of immune tolerance in patients with ITP being treated with romiplostim.

Surely, this cannot be the sole interaction of TPO-RAs with the underlying disease, since other TPO-RAs, such as eltrombopag seem also to induce sustained responses in patients with ITP. One possible explanation is that the elevation of the platelet mass may increase the level of transforming growth factor-β (TGF-β) in the blood and induce tolerance pathways. Platelets are known to contain the largest amount of TGF-β in the human body (43). TGF-β is a central cytokine in the regulation of the immune system by Tregs and the maturation of both Tregs and Th17 cells from undifferentiated CD4+ T cells (44, 45).

Studies addressing the tolerogenic activity of the Fc fragment in a given disease are rare. Pioneering studies included the extended half-life factor VIII- efmoroctocog alfa- (rFVIIIFc, elocta®) in hemophilia A patients (29). The main complication of factor replacement therapy in hemophilia A is the formation of inhibitors (neutralizing anti-factor VIII antibodies) rending replacement FVIII treatment essentially ineffective. The Fc domain of rFVIIIFc seems to confer immunomodulatory and anti-inflammatory properties in hemophiliac patients (46). Krishnamoorthy et al. showed a decreased immune response to rFVIIIFc products and induction of immune tolerance by rFVIIIFc in FVIII-deficient mice (21). In addition, an *in vitro* study showed that rFVIIIFc, but not rFVIII, uniquely skews macrophages toward an alternatively activated regulatory phenotype (46). The main potential benefit of rFVIIIFc compared to other FVIII preparations would be an increased tolerance toward factor VIII with less induction of inhibitors. Further studies are needed, including clinical research, to assess the real advantage of the Fc fragment.

The effects of TPO-RAs on the immune system are still being investigated, and several theories on the tolerogenic activity by these drugs have been offered (21). The effects of the Fc-Fragment of romiplostim have not been yet evaluated and is probably underestimated.

## Conclusion

TPO-RAs stimulate megakaryopoiesis and increase platelet count in ITP patients. Clinical and experimental research have shown that these drugs positively interact with the immune system and probably explain sustained remissions in nearly 30% of adult patients with ITP. Different theories are being investigated, and the platelet mass seems to play a central role. In the case of romiplostim the interaction of the Fc fragment with the immune system may be of potential value. Romiplostim belongs to the increasing group of Fc-fusion biotherapeutics and was designed and produced with the unique aim of prolonging the half-life of the active component. The study of Fc fusion proteins have and may reveal more tolerogenic properties of the “carrying” Fc molecule.

## Author Contributions

AS wrote the manuscript. AS and TK conceived the original idea. TK and FN provided critical feedback.

### Conflict of Interest Statement

AS: Amgen (research funds). TK: Novartis (research funds), Amgen (research funds), UCB biosciences (advisory board). The remaining author declares that the research was conducted in the absence of any commercial or financial relationships that could be construed as a potential conflict of interest.
